# Information needs and barriers to patient-centered care in oncology: EtnobreastHER2, an ethnographic study with HER2+ MBC patients and their healthcare providers

**DOI:** 10.3389/fmed.2025.1592380

**Published:** 2025-10-23

**Authors:** José Ángel García-Sáenz, Álvaro Rodríguez-Lescure, Silvia Antolin, Blanca Cantos, Miguel Ángel Seguí, María Antonia Gimón, Raül Rubio Renau, Carlota Solà Marsiñach, Jesús Martín Illán, Rocío Fonseca Vallejo, Yaiza Gimeno Guadalupe, Noelia Alfaro Oliver, Joaquín Gavilá

**Affiliations:** ^1^Oncology Department, Hospital Ruber Internacional, Madrid, Spain; ^2^Department of Medical Oncology, University General Hospital of Elche, Elche, Spain; ^3^Oncology Department, A Coruña University Hospital Complex (CHUAC), A Coruña, Spain; ^4^Department of Medical Oncology, Puerta de Hierro University Hospital Majadahonda, Majadahonda, Spain; ^5^Oncology Department, Parc Taulí University Hospital, Sabadell, Spain; ^6^Spanish Federation of Breast Cancer FECMA, Presidency, Madrid, Spain; ^7^Evidence Generation Department, A Piece of Pie, Barcelona, Spain; ^8^Medical Department, Daiichi Sankyo Spain, Madrid, Spain; ^9^Medical Department, AstraZeneca Farmacéutica Spain, Madrid, Spain; ^10^Oncology Department, Instituto Valenciano de Oncologia, Valencia, Spain

**Keywords:** human epidermal growth factor receptor 2 metastatic breast cancer, oncology, patient-centered care, informational needs, doctor-patient communication, structural barriers

## Abstract

**Introduction and objectives:**

Qualitative evidence regarding the structural barriers embedded in healthcare ecosystems impacting Human Epidermal Growth Factor Receptor 2 metastatic breast cancer (HER2+ MBC) patients’ communication and information needs is scarce. This study explored patient and healthcare professionals’ perspectives on said structural barriers and communication delivery.

**Methods:**

Ethnographic, qualitative, observational, multicenter and cross-sectional study with HER2+ MBC patients and Health Care Professionals. Qualitative data collected through remote semi-structured interviews with patients (*n* = 14) and healthcare professionals (*n* = 10). The European Organization for Research and Treatment of Cancer Quality of Life Questionnaire (EORTC QLQ)-C30 and the disease-specific EORTC QLQ-BR45 questionnaires were collected as Quality of Life measures.

**Results:**

Regarding the communication and information needs of patients, there were four areas these were lacking most: (1) disease and treatment, (2) psychosexual support, (3) navigation of the healthcare and social security systems to reduce the financial burdens, and (4) patient associations. In reference to delivery of information from healthcare professionals, there were four areas that were lacking: (1) time, (2) interpersonal skills and communications training, (3) specialized oncology nursing training, and (4) lack of an evaluation system assessing patient-centered care and patient satisfaction.

**Conclusion:**

Results emphasize how structural barriers embedded in healthcare systems can lead to and intensify information and communication gaps, which therefore emphasizes that to provide optimal, patient-centric care, these structural barriers must be addressed.

## 1 Introduction

Breast cancer (BC) remains a significant and highly prevalent cancer, accounting for 24.5% of all cancer cases and 15.5% of all cancer related mortalities worldwide (1, 2). Projections suggest a 31% increase in new BC cases by 2040, with mortality rates expected to rise due to population growth and aging (1). In Spain, BC accounts for 12% of all diagnosed cancers, and around 5–6% of cases are already metastasized at the time of diagnosis (3). The human epidermal growth factor receptor 2 (HER2) is a tyrosine kinase receptor that signals to promote several growth-related pathways. BC that overexpress this receptor are classified as HER2-positive (HER2+), accounting for 15–20% of all BC cases worldwide (4–6). In Spain, early estimates approximate that HER2+/Hormone Receptor-positive (HR+) and HER2+/HR-negative (HR-) account for 16% and 9.4% BC cases, respectively (7). HER2+/HR- BC patients experience more aggressive disease and worse outcomes than HR+ patients, requiring targeted anti-HER2 therapies with chemotherapy, and endocrine therapy (8). These treatments have improved patient survival rates in recent years and they often require long-term care (9, 10).

This study is situated within the Spanish context, where a publicly funded National Health System operates. The system is structured as a decentralized health network governed by the Autonomous Communities and implements integrated cancer care strategies aligned with the national Cancer Strategy 2006–2014 (11). In 2024, 36.395 new BC cases were diagnosed in Spain, representing the most common cancer among women (12). Although the patient experience of metastatic BC has been only briefly explored in Spain, one qualitative study mapping BC patient journeys identified significant gaps in emotional and social support among patients at an incurable stage, highlighting the need to implement a stronger psychological care approach (13) Limited access to new therapies is also an area of concern in the Spanish context, since the country experiences longer delays than most EU countries between drug approval from the European Medicines Agency and reimbursement from the health system, a necessary condition for prescription (14). This delay may negatively impact patients’ treatment trajectories and increase the psychosocial burden associated with BC. Several studies have reported on the unmet needs of patients with BC and, to a lesser extent, HER2+ metastatic breast cancer (MBC). These highlight impacts on physical/daily life, psychological impacts, symptoms burden, and patient care, as well as communication and information needs (15–20). Given that patient-centric communication is increasingly being recognized to positively impact treatment adherence, quality of care, management of chronic disease and other health outcomes (21), there is a significant amount of literature discussing the information needs of oncological patients more generally, and BC patients in particular (17, 22–25). This literature is frequently paired with recommendations to improve doctor-patient communication, asking healthcare professionals (HCPs) to be mindful of the emotional and psychosocial needs of patients, and so on (26–28). This is considered essential for patients struggling to cope with parts of their journey, such as diagnosis, treatment, and the impact on inter-personal relationships and social engagements (26–28).

However, there is a relative dearth of research into the constraints faced by HCPs within the healthcare system and how they impact information delivery. Notable constraints include geographical limitations, cost restrictions, inadequate HCP training, and time pressures (29–35). Acknowledging these barriers is essential, since addressing patients’ information needs without considering the characteristics of the healthcare system places unrealistic expectations on HCPs.

To address this gap, this paper presents the perspectives of both patients and HCPs. It gives voice to women with HER2+ MBC, illustrating how HCPs could enhance communication delivery from the patients’ viewpoint. Additionally, it outlines the structural barriers faced by oncologists and nurses when it comes to addressing communication delivery. We focus on patients with a diagnosis of HER2+ stage IV BC, a unique clinical subgroup characterized both by therapeutic advances and ongoing challenges. The appearance of targeted therapies in recent years has significantly improved disease outcomes and survival rates (36). Nevertheless, HER2+ stage IV BC remains incurable, situating patients in a complex clinical and emotional situation which is different from early-stage diagnosis (37). This clinical scenario gives rise to specific complexities related to patients’ informational needs, mainly because they face prognostic uncertainty and navigate the blurry boundaries between palliative and chronic care, as we have shown in a separate publication (57).

## 2 Patients and methods

### 2.1 Study design

The EtnobreastHER2 Study is an ethnographic, qualitative, observational, multicenter and cross-sectional study that aimed to recruit 20 patients with HER2+ MBC, five oncologists specialized in the treatment of BC and five nurses routinely seeing patients with BC from five university hospitals in Spain. Patients were recruited face-to-face during their routine visits. After obtaining informed consent, doctors compiled information related to patients’ personal characteristics, disease and treatment. This contact information was shared with an anthropologist, who scheduled and conducted the interviews with patients. The interviewer was female, white and Spanish, and had 10 years of experience conducting interviews with patients in the medical field. Interview questions were open-ended and aimed to elucidate the lived experience of HER2+ MBC patients. HCPs were recruited by the principal investigators of the study from the participating hospitals. HCPs were interviewed after almost all patient interviews were conducted, with the aim of complementing their view. Interview questions were also open-ended but covered the themes identified in patient interviews.

### 2.2 Sample and recruitment

All patients to be included in the study had to have a documented diagnosis of locally advanced or HER2+ MBC, be receiving second line treatment according to SEOM (Spanish Society of Medical Oncology) clinical guidelines after having progressed from first line treatment at the time of the inclusion and be ≥ 18 years at the time of consent. Participating in a clinical trial, being hospitalized at the time of inclusion, having been previously diagnosed with cancer, being under treatment for other types of cancer, or holding a managing position in a patient association were all exclusion criteria. Participants were recruited consecutively. Of the 17 patients who initially agreed to participate, three withdrew their consent: two of them stopped responding to requests to participate without providing any reason, and one responded she did not feel well enough to participate in an interview. Patients who agreed to participate were recruited from four university hospitals in Spain. All 10 HCPs had to routinely see patients with BC. Participating HCPs were four oncologists and six nurses.

Literature discussing sample sizes in qualitative research estimate that a sample size of 10 or more is considered sufficient to reach empirical saturation –the point in which increasing the number of observations does not provide new data– in ethnographic studies (38). With a sample size of 24 participants, our study falls within the recommended guidelines and allowed us to reach saturation.

### 2.3 Data gathering and analysis

Patients and HCPs participated in semi-structured interviews –2h long for patients and 90-min long for HCPs–. Interviews were aimed to be carried out in-person but were allowed to be performed remotely if local healthcare authorities or medical centers recommended preventive measures against covid-19, or if the patient preferred so. In such cases, remote interviews were to be performed using encrypted tools such as Zoom, Teams or Skype to ensure the protection of patients’ private data. Eventually, all interviews were conducted remotely via Teams, following participants’ preferences. Semi-structured interviews allowed participants to raise issues of concern while simultaneously covering the researcher’s areas of interest, and only the participant and the interviewer were present. There was no relationship between the interviewer and participants prior to the study. At the beginning of the interview, patients were informed of the interviewer’s position and the research goals. The study was neither pilot tested nor were repeat interviews conducted. Fieldnotes were made during and after the interview to complement the data collected, which was used in the analysis for this paper. Transcripts were not returned to participants for comment or correction, and they did not provide feedback on the findings.

Additionally, patients completed two patient-reported outcome measures (PROMs) at the beginning of the interviews in the presence of the ethnographer but without intervention. These were the generic European Organization for Research and Treatment of Cancer Quality of Life Questionnaire (EORTC QLQ-C30) and the disease-specific EORTC QLQ-BR45 questionnaires. PROMs were used as a conversation starter, to approach sensitive topics, and to prompt patients to self-reflect on their condition without the intervention of the ethnographer. The information gathered with these PROMs was analyzed descriptively.

Two anthropologists independently coded all fieldwork materials following a phenomenological perspective, which aims to elucidate the lived experience of a disease and the conditions shaping said experience (39, 40). The analytical process was both inductive and deductive, meaning that themes were identified by topics emerging directly from the data (inductive inference) and applying prior knowledge (deductive inference) (41). Only themes which reached empirical saturation, the point at which new data no longer emerge, were selected for analysis (42, 43). A vast amount of themes emerging from the EtnobreastHER2 Study reached saturation. To be able to present them in depth, this study solely focuses on those related to information needs and barriers to patient-centered care. Additionally, there is a description of minor themes in the results and discussion parts of this article.

## 3 Results

### 3.1 Participant characteristics

The final sample consisted of 24 participants: 14 patients, four oncologists and six nurses. All patients interviewed had stage IV HER2+ BC and were receiving second line treatment after having progressed from a first line. Patients’ mean age was 53,5 years, and their mean time since diagnosis was 5,2 years. HCPs were 80% female. Participants’ demographic and clinical characteristics are compiled in Table 1.

**Table 1:** Participant characteristics.

Patients	Demographic and clinical data	*n*	%
Gender	Female	14	100
Age (mean)	53,5	14	
Age (range)	18–34	0	
	35–59	11	
	≥ 60	3	
Time since HER2+ MBC diagnosis (mean)	5,2	14	
Time since HER2+ MBC diagnosis (range)	2020–2023	7	50%
	2016–2019	3	21,42%
	Before 2016	4	28,57%
Second line treatment	TDM1	8	57,14%
	Trastuzumab-deruxtecan	6	42,85%
Disease stage	IV	14	
Marital status	Single	1	7,14%
	Married	10	71,42%
	widowed	3	21,42%

HCPs		*n*	%
Profession	Oncologist	4	40%
	Nurse	6	60%
Gender	Male	2	20%
	Female	8	80%

### 3.2 Delivery of patient-centric communication: the patients’ perspective

Patients in our study identified several areas of improvement in the delivery of patient-centric communication for HER2+ MBC. Illustrative quotes on this topic can be found in Table 2.

**Table 2:** Delivery of patient-centric communication: the patients’ perspective.

More information about the disease and treatments	I. You have to find the information yourself, and sometimes you just cannot get it. (45-year-old patient with stage IV HER2+ BC) II. There should be someone to help you with the doubts and emotional states you go through during the process. Someone you could call and explain your problem to, like a primary care psychologist. Someone who could explain the medications they are giving you, for example. ‘Why are they giving me this now?’ They’re giving me biological injections, but I really do not know what that is or what it’s for. (63-year-old patient with stage IV HER2+ BC)
More information about psychosexual support	III. At the beginning, when you are diagnosed and start treatment, sex takes a back seat. But it would be helpful to receive sexual support during the disease, because it can cause issues with your partner. For example, I experience a lot of dryness (45-year-old patient with stage IV HER2+ BC) IV. I’ve never received any help regarding sexuality. It would have been nice if someone had brought it up. (50-year-old patient with stage IV HER2+ BC)
Practical support to navigate the healthcare system	V. I find it very frustrating that the doctors did not tell me about the financial assistance available. I have to travel 60 km to get to the hospital, and the doctors never mentioned that I could request financial aid. It was a friend from my town, who had the same condition, who had to tell me. (51-year-old patient with stage IV HER2+ BC)
More information about PAs	VI. I looked into it, but for older women it can be harder, and they may not get informed and might not receive the right assistance. The information [about the associations] is posted on pamphlets in the rooms where they administer the medication. But, of course, if a nurse told you, it would carry more weight than just being stuck on a poster. (45-year-old patient with stage IV HER2+ BC)

First, eight patients (57%) were not satisfied with the amount and kind of information received regarding the disease and treatments. Some preferred to have more time to ask questions and lengthier explanations, while others preferred to receive simpler explanations with less medical jargon. Reasons for this included: (a) not understanding the information provided and (b) not being able to properly transmit to their caregivers the seriousness of their condition. As a result of point (a), patients stopped asking questions, anticipating they would not understand HCPs’ answers, and looked for information online. As a result of point (b), patients’ sense of being misunderstood and unsupported by their social circles increased, thus negatively impacting their mood (Table 2; verbatims I-II).

Second, eight patients (57%) wanted to receive more information on psychosexual support. Most patients explained not being interested in sexual activity (Table 2; verbatims III-IV). Due to their loss of libido and decreased engagement in sexual activity, some women felt they could not satisfy their partners, with some fearing their partners would leave them. Others felt that being sexually active and enjoying sexual activity was part of being healthy and returning to normalcy and therefore strived to achieve it. The descriptive analysis of the EORTC QLQ-BR45 showed that more than half of the women were ‘not at all interested’ in sex (eight), six were only ‘a little interested,’ and none were ‘quite a bit interested’ or ‘very much interested.’ Furthermore, nine were ‘not at all sexually active,’ five were ‘a little active,’ and none were ‘quite a bit’ or ‘very much active’. From those who were sexually active, none did ‘not enjoy sexual activity,’ five ‘enjoyed it a little,’ and none ‘enjoyed it quite a bit’ or ‘enjoyed it very much.’ PROM results regarding this topic can be found in Table 3.

**Table 3:** Responses to EORTC QLQ-BR45 questions on sexuality.

During the past 4 weeks:			
Measures	Interested in sex	Sexually active	Enjoyment of sex
Obs	13	13	11
T2B	0%	0%	0%
Mean	1.5	1,4	1,5
StDev	0,5	0,5	0,5

N, Number of cases. T2B, The sum of the two highest frequencies (answers 3 and 4). Mean, Arithmetic average. StDev, Standard Deviation. Measurement scale: 1–4, with 1 being the lowest (‘not at all’) and 4 being the highest (‘very much’).

Third, eight patients (57%) wanted to receive practical support on how to navigate the healthcare and social security systems to reduce the financial burden the disease had on themselves and their families. Some patients would have liked their HCPs to recommend resources where to obtain wigs and creams for free if possible, or at a reasonable price. Others would have liked their HCPs to draw attention to social security services or non-governmental organizations (NGOs) that could help with their homecare or transportation needs, especially when they had to travel long distances to access care. These patients had found in retrospect, via friends or acquaintances, that such services existed and that they could benefit from them (Table 2; verbatim V).

Fourth, seven patients (50%) wanted to receive more information about patient associations (PAs). Some patients were members of PAs and benefited from sharing their experiences with other patients. However, they had heard about said groups from their acquaintances, when they would have rather heard about them from their HCPs (Table 2; verbatim VI). Other patients did not want to socialize with other patients, arguing that they did not feel emotionally prepared to face others in similar circumstances. Nevertheless, many of these patients were unaware that socializing with other patients is not the only role provided by PAs; in retrospect, they found out about the resources provided by these groups, such as psychological support and practical resources to improve QoL, such as discounted sports activities and social aid. These patients said they would have appreciated information from their HCPs clarifying this role of PAs.

### 3.3 Doctor-patient communication: the HCPs’ perspective

HCPs were mostly aware of the perspective of the patients regarding information delivery. All 10 HCPs considered important to address it to improve patient-centered care. However, they argued that there were structural barriers hindering the process. Illustrative quotes regarding this topic can be found in Table 4.

**Table 4:** Delivery of patient-centric communication: the HCPs’ perspective.

Lack of time	I. Because in our practice, we have such a heavy workload that, even if we wanted to, there’s no time. If you have between 20 and 25 patients in a day, and each one needs more information or more time than the 5 or 10 min you have to check that everything is okay, it can get complicated. […] Lack of time, the stress of getting patients in on time, ensuring they do not wait too long in the waiting room, and hoping that most of them do not need more time than necessary that day. (Female oncologist) II. And the number of patients has increased so much, with such a large volume of patients in the practice, that sometimes there are 30 patients to see in one morning. With 30 patients, you just do not have the time to explain everything in detail. Another challenge is how well the person understands the information. Some people might get it the first time, but others need you to explain it two or three more times. That requires more time–time we simply do not have. (Female nurse) III. I firmly believe that there is indeed never enough time for all the patients—this is a reality. But I also think that investing time in the beginning can save a lot of time later on. (Female nurse)
Lack of training on interpersonal skills and communication	IV. I try to get my residents to take courses on how to deliver bad news, but it’s not formalized. The Spanish Society offers a course for younger residents so they can learn about these things. However, there’s no specific training on this. I try to ensure that my residents get more than just training—I want them to develop values. But it’s complicated because what matters most is the résumé, publications, articles… and in the end, not everyone is prepared to be a good doctor. […] What we try to challenge is the idea that doctors should only have good grades. That’s still an unresolved issue in the Western healthcare system. (Male oncologist) V. I believe that nowadays, oncology specialists and residents have a bit more training, or at least there’s an effort to ensure that they learn communication techniques during their specialization. But this is still an issue that universities have yet to fully address, starting right there. It’s something that is not valued enough in medical education. This happens in all areas of medicine, not just oncology, even though we are the ones who deliver bad news the most. I think it’s essential, and it’s also an unmet need in our training. (Female oncologist)
Lack of specialized oncology training	VI. Yes, I believe we need training for nurses who are passionate about oncology and work in the field. (Female oncologist) VII. Since there is not a specific oncology specialization, you are left lacking the necessary knowledge to work in oncology. All the master’s programs I’ve done, I had to pay for myself. I’ve completed several master’s programs, but they did not really work out for me, because as soon as I finished or was in the middle of one, I would be moved out of the unit, and then the degree became useless. That’s why I ultimately decided to pursue research, because I did not know what specialization I would end up with. (Female nurse)
Lack of patient-centered evaluation system	VIII. No one pays attention to whether you have good reviews. Sure, you have an interview with your boss, but no one bothers to ask how you are with the patients. And if a position opens up, the person with the best résumé would win, not the one who is working hard every day and putting in the effort. I’ve found that to be very unfair, and it’s made me cry. (Female nurse) IX. What they value are your achievements, what you have published, written, but the truth is that the human accomplishments, the way you care for others, remain largely between us. There’s no place to document that—it’s all through word of mouth. (Female nurse) X. In oncology, you are only valued for what you publish, the talks you attend, and the ones you give. Your scientific résumé carries much more weight than your clinical experience. No one asks how many patients you see, or what your patients’ survival rates are—that’s never analyzed. […] They only look at the number of publications, and if you are a clinician seeing patients, you have to take that time out of your personal life. (Female oncologist)

The first barrier, identified by all 10 HCPs (100%), was the lack of time to dedicate to each of their patients due to an overburdened healthcare system. This precluded the possibility to provide more information regarding all the unresolved issues raised by patients (Table 4; verbatims I-III). Several HCPs reported that they usually lacked sufficient time to provide detailed explanations during consultations. The average duration of five to 10 min per patient was described as insufficient for thoroughly addressing all patients’ questions or providing more comprehensive explanations, particularly to those who struggled to understand medical information. This limitation was also identified in relation to psychosexual health, a topic that patients rarely raised spontaneously and, as a result, was often overlooked during medical visits. All HCPs emphasized the negative impact that limited time during consultations had on patients’ satisfaction.

The second barrier, identified by nine HCPs (90%), was a lack of training in interpersonal skills and the communicative aspects of the profession. HCPs emphasized that training on adequate communication, delivery of bad news, and so on, would help them address the needs of patients. HCPs emphasized their lack of training to be an emotional pillar in times of need, highlighting that the relevant skills were acquired with practice and trial and error. These left young HCPs, and young nurses in particular, feeling inadequate in their ability to emotionally support their patients (Table 4; verbatims IV-V). This difficulty was also evident in the limited information provided to patients regarding PAs, as highlighted in patients’ accounts. HCPs considered they often lacked enough knowledge about the specific forms of support PAs could offer. According to their accounts, specific training programs for professionals should be implemented to enhance their understanding of the support offered by PAs, as well as the existing financial assistance and public subsidies available to patients.

The third barrier, identified by seven HCPs (70%), was a lack of specialized oncology training in nursing. As reported by HCPs, there is no formal specialization in oncology in the five-year nursing degree in Spain. In order to specialize in oncology, nurses can either undertake self-funded specialized courses or master’s degrees or obtain on-the-job experience while in a position in an oncology department after graduation. Both courses of action were presented by nurses as difficult to attain, since a position in an oncology department was never guaranteed due to the frequency of rotation between posts and specializations. As a result, nurses explained that they generally did not invest in specialized oncology courses. According to HCPs, lack of oncology nursing specialization may lead to suboptimal care for oncology patients. Overall, their lack of knowledge hindered them from supporting oncologists in the delivery of information and the provision of emotional and practical support (Table 4; verbatims VI-VII). More in particular, the lack of specialized oncology training for nurses was identified as a missed opportunity to address certain patient needs that oncologists were unable to meet due to the barriers explained above. Nurses reported not having enough preparation to advise patients on the impact of the illness on their sexual lives, hair loss, or skin care. They also expressed feeling unprepared to effectively guide patients through the complexities of the healthcare system and emphasized the need for a specific training program to address these gaps.

The fourth barrier, identified by six HCPs (60%), was the lack of an evaluation system assessing patient-centered care and patient satisfaction. According to HCPs, the current evaluation system is focused on congress attendance and publications, and rewards only those HCPs who excel at these rather than at delivering patient-centered care (Table 4; verbatims VIII-X). In this sense, demonstrating empathy and communicating effectively with patients were perceived as an “invisible” effort, and therefore, could fail to motivate HCPs to develop a better understanding of how to support patients beyond strictly clinical follow-up. In addition, the use of patient questionnaires was identified as a helpful tool that could be implemented to facilitate conversations about sexuality, a topic that HCPs perceived as taboo for numerous patients.

## 4 Discussion and conclusion

### 4.1 Discussion

The results of this paper identified areas of improvement in oncologists’ and nurses’ communication delivery from the perspectives of both HER2+ MBC patients and their treating physicians, while at the same time highlighting the structural barriers hindering the provision of said communication delivery from the perspective of HCPs.

There are several studies discussing the communication needs of oncological patients more generally, and BC patients in particular. Many draw attention to patients’ information needs regarding the disease and treatments and to the need for psychosocial support (17–25). For patients with MBC, there are many areas where patients are lacking information. Examples include symptoms of metastases, treatment options, side effects, pain management, clinical trials, immunotherapy, acupuncture, social security assistance, and types of treatment, all of which have been shown to relate to increased levels of anxiety, depression, sexual difficulties, self-image, and pain (44–46). Patients who are living with HER2+ MBC also have a gap in communication in supporting physical, emotional and psychosocial concerns that result in tiredness, decreased sexual interest, less satisfaction with relationships, sore muscles, anxiety, difficulty sleeping, and joint pain (47, 48). Resonating with the literature, patients in our study agreed with the need to receive more information on the disease, treatments, and psychosexual support, confirming these needs for the Spanish context. Furthermore, they provided granularity to identified needs for social security assistance and social support with the necessity to obtain resources such as accessibility to transportation, access to charities, knowledge on PAs, and reasonably priced creams, wigs, and so on. To increase patient-centered information delivery, these needs should be addressed in communication encounters between HER2+ MBC patients and their oncologists and nurses. Additionally, having a dedicated support staff to ensure this information is provided throughout the journey could alleviate the negative impact of not receiving this information on overall mood.

Unfortunately, studies reporting on information needs of oncological patients tend to not consider the structural barriers that the healthcare system imposes on HCPs. For instance, a review meta-analysis conducted by Sisk and colleagues shows that out of 109 studies on communication needs of cancer patients, the vast majority focused on individual-level barriers rather than team, organization/system, collaborating hospital, community, or policy-level barriers (22). Therefore, a common conclusion of these type of studies is to make recommendations that appeal to HCPs as individuals (17, 20, 21, 49). Examples of these include asking HCPs to dedicate more time and energy to providing patient-centered information, or asking them to assess and address the psychosocial wellbeing of patients (17, 20, 21, 49).

Of the few studies that do address the structural barriers faced during the communication encounters in oncology, most of them discuss low and middle income countries (LMICs) (50–53). For instance, Paz-Soldán and colleagues conducted a qualitative study in Peru drawing attention to the structural barriers to screening for and treatment of cervical cancer in the country (50). In LMICs there are numerous structural barriers that women face in accessing healthcare services for breast cancer. These can range from issues of accessibility due to geographical and infrastructural challenges, costs associated with screening, and healthcare system challenges, such as inadequate HCP trainings and a lack of culturally competent medical care (29–32). All of these challenges can make women feel isolated and unsupported in clinical settings.

Instead, studies addressing structural barriers to communication delivery between HCPs and oncology patients in high income countries are scarce. Dencker et al. conducted a qualitative study in Denmark with doctors and nurses working with patients with gynecological and hematological cancers and in neurointensive care to identify the barriers hindering HCPs from communicating with patients about their children. They identified several emotional and structural barriers, the latter being ‘lack of space in the medical recording system,’ ‘professional code,’ ‘time pressure,’ and ‘lack of training.’ (33) Haussmann et al. analyzed structural barriers to promoting physical activity to cancer patients among physicians and nurses in Germany, and found that ‘not enough time per patient’ was the most cited barrier (34). In addition to HCPs’ workload, timing, coordination, information material for HCPs and patients, and availability of exercise programs were all identified as structural barriers to the promotion of physical activity (35).

Our study adds to this body of literature for patients with HER2+ MBC and their treating oncologists and nurses in Spain. HCPs in our study were perfectly aware of the potential room for improvement in their communicative encounters with patients. They identified lack of time as the main structural barrier hindering optimal communication with them, coinciding with the studies of Dencker et al. and Haussmann et al., while lack of training on interpersonal skills and communication was identified in both our study and that of Dencker et al. Furthermore, HCPs in our study presented two new structural barriers to communication not identified in the literature: having an evaluation system not assessing patient-centered care and patient satisfaction and lacking a specialized oncology training in nursing.

The figure of the specialized oncology nurse, present in other countries, has been found to be essential in the provision of patient-centric communication (54, 55). Specifically, studies have identified the need to empower oncology nurses to recognize emotional distress in their patients, as it is ‘the sixth vital sign which should regularly be monitored’ (27, 28). Moreover, research has identified that the lack of education on patients’ mental health, particularly on how to evaluate this, results in disregarding emotional states as part of their duty of care (28, 56). This paper agrees with these findings, arguing that specialized oncology nurses would be better prepared to share with oncologists the burden of information delivery regarding the topics identified in this study.

Although certain sections of our interview guides were specifically designed to explore the experiences of HER2+ patients, we believe that the findings of this study are applicable to the general BC population. Informational needs and barriers to access and understand medical information are challenges faced by patients across all BC subtypes, not exclusively those with HER2+ disease.

#### 4.1.1 Strengths and limitations

A limitation of this study is the small sample size of 14 patients recruited at five university hospitals, which may not represent the full diversity of people diagnosed with stage IV HER2+ BC. A further limitation is that the study is cross-sectional with a single interview per patient, not providing patient perceptions as their disease evolves. However, the fact that participants correspond to a relatively homogenous population provides a compelling picture of the disease. Furthermore, the interviews with oncologists and nurses have been used to validate and complement patients’ insights.

### 4.2 Conclusion

This study has identified HER2+ MBC patients’ perspectives about the communication delivery they receive from their HCPs regarding the disease and treatments, psychosexual support, navigating the healthcare and social security systems, and PAs. This particular group of patients was selected for the study based on the specificities of their clinical and emotional experience. Living with a disease that is treatable yet incurable, they are confronted with the practical and conceptual ambiguity of navigating chronic and palliative models of care. In the specific Spanish context, improved psychological support and more tailored information provision have been identified as two key areas for better addressing the needs of these patients (13, 38). By also including the voices of HCPs, we have provided a wholistic, nuanced perspective on the gap between patient information needs and HCPs capacities and have demonstrated how these become magnified by inadequate healthcare ecosystems.

The deepened appreciation of how a healthcare system can pressurize on HCPs and lead to inadequate support can help policy makers and medical educators to tailor programs to the real-world needs of HCPs. In turn, they would be better equipped to support their patients through a challenging physical and emotional patient journey, which could result in improved patient outcomes and experiences.

To provide optimal, patient-centric care for HER2+ MBC patients, we conclude that individual level improvements are insufficient, and that it is paramount to consider and address the structural barriers imposed on HCP care delivery by the Spanish healthcare system.

### 4.3 Implications for practice

This article highlights the specific issues encountered by patients during interactions with their doctors and nurses. We consider these insights to be a powerful tool with the potential to inform the improvement of policies and protocols within the healthcare system and administration. Regarding the difficulties evoked by professionals, the lack of oncological specialization for nurses only represents a minor aspect of a broader issue. More generally, it is necessary to work on the improvement of communication channels and joint work between nurses and oncologists. This collaboration would have a positive impact on patients’ experiences.

## Data Availability

The datasets presented in this article are not readily available because the data underlying this article cannot be shared publicly due to the privacy of individuals that participated in the study, as established by the ethics committee. The data underlying this article will be shared upon reasonable request to the corresponding author. Requests to access the datasets should be directed to jagsaenz@yahoo.com.
